# Growth Differentiation Factor 11 treatment leads to neuronal and vascular improvements in the hippocampus of aged mice

**DOI:** 10.1038/s41598-018-35716-6

**Published:** 2018-11-23

**Authors:** Ceren Ozek, Richard C. Krolewski, Sean M. Buchanan, Lee L. Rubin

**Affiliations:** 1 Department of Stem Cell and Regenerative Biology, Harvard University, Cambridge, MA 02138 USA; 2 Harvard Stem Cell Institute, Harvard University, Cambridge, MA 02138 USA; 3 Department of Neurology, Brigham and Women’s Hospital, Massachusetts General Hospital, Boston, MA 02115 USA

## Abstract

Aging is the biggest risk factor for several neurodegenerative diseases. Parabiosis experiments have established that old mouse brains are improved by exposure to young mouse blood. Previously, our lab showed that delivery of Growth Differentiation Factor 11 (GDF11) to the bloodstream increases the number of neural stem cells and positively affects vasculature in the subventricular zone of old mice. Our new study demonstrates that GDF11 enhances hippocampal neurogenesis, improves vasculature and increases markers of neuronal activity and plasticity in the hippocampus and cortex of old mice. Our experiments also demonstrate that systemically delivered GDF11, rather than crossing the blood brain barrier, exerts at least some of its effects by acting on brain endothelial cells. Thus, by targeting the cerebral vasculature, GDF11 has a very different mechanism from that of previously studied circulating factors acting to improve central nervous system (CNS) function without entering the CNS.

## Introduction

Adult neurogenesis, the process by which new functional neurons are generated and integrated into existing neuronal circuits of the adult brain, occurs in two specific regions of the mouse central nervous system (CNS): the subgranular zone (SGZ) of the hippocampus and the subventricular zone (SVZ)^1^. In both brain regions, neurogenesis occurs in a niche where neural stem cells reside near blood vessels. Signals from neural cells, as well as from the vasculature, influence neural stem cell proliferation and differentiation^2,3^. Neurogenesis is known to be regulated by a variety of stimuli. For example, exercise is a positive regulator of neurogenesis, while stress is a negative regulator^4^. Aging is also a negative regulator of neurogenesis and is associated with decline in the number of neural stem cells and their differentiation^5,6^. Aging also results in impairments in structural and functional aspects of the cerebral vasculature through reduced vascular density and blood flow^7,8^.

Heterochronic parabiosis, through which systemic factors circulating in young and old mouse blood are shared, positively influences neurogenesis, cerebral vasculature, neuronal activity, synaptic plasticity and cognitive function in old mice^9–11^. Several individual circulating factors, some having positive actions, some negative, have already been identified^12–14^. A recent study from our lab demonstrated that systemic treatment with one of them, Growth Differentiation Factor 11 (GDF11), a member of the Transforming Growth Factor beta (TGFβ) superfamily of proteins, had positive effects on old mouse brain^11^. Notably, GDF11 treatment increased the number of neural stem cells and blood vessel density in the SVZ of old mice. Furthermore, genetic activation of the activin-like kinase 5 (ALK5) receptor that binds GDF11, as well as related ligands, and activates downstream signaling through Sma- and Mad-related proteins 2/3 (SMAD2/3) improved neurogenesis, neuronal activity, synaptic plasticity and cognition in the hippocampus of old mice^15^.

The hippocampus has been studied extensively for age-related structural and functional impairments as well as age-dependent deficits in learning, memory and cognition^16^. Additionally, the hippocampus has been implicated as one of the most functionally significant structures affected by neurodegenerative and neurovascular diseases since hippocampal deficits are associated with declining cognitive ability^17^. Although our previous study showed beneficial effects in the SVZ, whether systemic GDF11 treatment exerts similar effects on hippocampal neurogenesis and vasculature remained unknown. In this study, we extend our previous findings and demonstrate that systemic GDF11 treatment enhances neurogenesis, improves vasculature, and increases the expression of neuronal activity markers in the hippocampus of old mice. We also provide evidence that GDF11 does not cross the blood brain barrier (BBB) and that the endothelial cells of the cerebral vasculature are responsive to GDF11, suggesting that GDF11 exerts at least a portion of its CNS effects through the vasculature. This distinguishes GDF11 from other individual circulating factors that have been shown to modulate aging in the brain by entering the CNS and acting directly on neural cells^4^. GDF11 may then be a novel rejuvenating factor that acts on vasculature within and outside of neurogenic brain regions.

## Results

### Systemic GDF11 treatment enhances neurogenesis in the hippocampus of old mice

**Figure 1:**
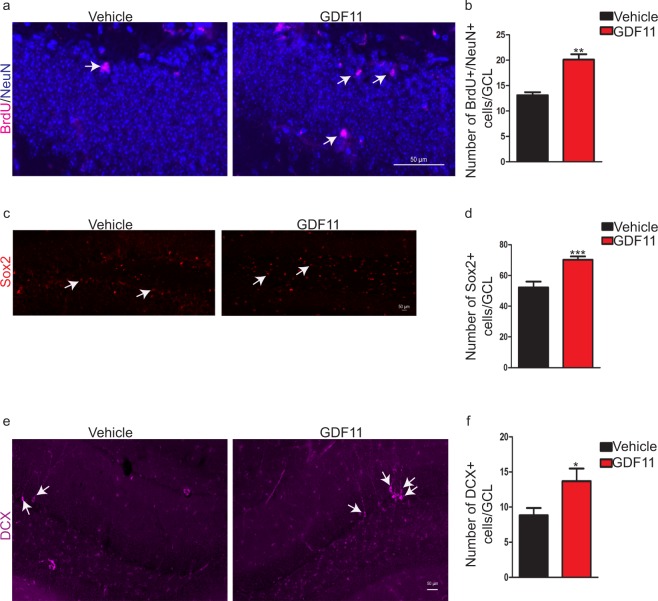
Systemic GDF11 treatment enhances neurogenesis in the hippocampus of old mice. (**a**) Representative confocal images showing the effects of systemic GDF11 treatment on BrdU^+^/NeuN^+^ newborn neurons in the GCL of old mice. White arrows indicate representative cells that are positive for both markers. (**b**) Quantification of BrdU^+^/NeuN^+^ newborn neurons in GCL (total area). n = 4 for each experimental group. Data shown as mean ± s.e.m., statistical analysis by unpaired, two-tailed Student’s *t-*test, **p = 0.005 compared to vehicle control. (**c**) Representative confocal images showing the effects of systemic GDF11 treatment on Sox2^+^ Type1 neural stem cells in the GCL of old mice. White arrows indicate representative cells that are positive for the marker. (**d**) Quantification of Sox2^+^ Type1 neural stem cells in GCL (total area). n = 8 for each experimental group. Data shown as mean ± s.e.m., statistical analysis by unpaired, two-tailed Student’s *t-*test, ***p = 0.001 compared to vehicle control. (**e**) Representative confocal images showing the effects of systemic GDF11 treatment on DCX^+^ neural progenitor/immature neurons in the GCL of old mice. White arrows indicate representative cells that are positive for the marker. (**f**) Quantification of DCX^+^ neural progenitor/immature neurons in GCL (total area). n = 8 for each experimental group. Data shown as mean ± s.e.m., statistical analysis by unpaired, two-tailed Student’s *t-*test, *p = 0.03 compared to vehicle control.

To determine whether systemic GDF11 treatment has beneficial effects on neurogenesis in the hippocampus of old mice, 22–23-month-old mice received daily intraperitoneal (i.p.) injections of GDF11 or vehicle for 28 days. As aging causes a decline in hippocampal neurogenesis^5^, we investigated whether this treatment could increase the number of newborn neurons, neural stem cells or neural progenitors/immature neurons in the hippocampus of old mice^18^. We found that GDF11 increased the number of BrdU^+^/NeuN^+^ newborn neurons (Fig. 1a,b), Sox2^+^ Type1 neural stem cells (Fig. 1c,d), and DCX^+^ neural progenitors/immature neurons (Fig. 1e,f) in the dentate gyrus. To assess whether neurogenic effects of systemic GDF11 treatment are also observed in young brains, 2–3-month-old mice received daily i.p. injections of GDF11 or vehicle for 28 days. Notably, GDF11 did not significantly change the number of neural progenitors/immature neurons (Supplementary Fig. S1a,b) in the dentate gyrus of young mice.

### Systemic GDF11 treatment improves vasculature in the hippocampus and cortex of old mice

**Figure 2:**
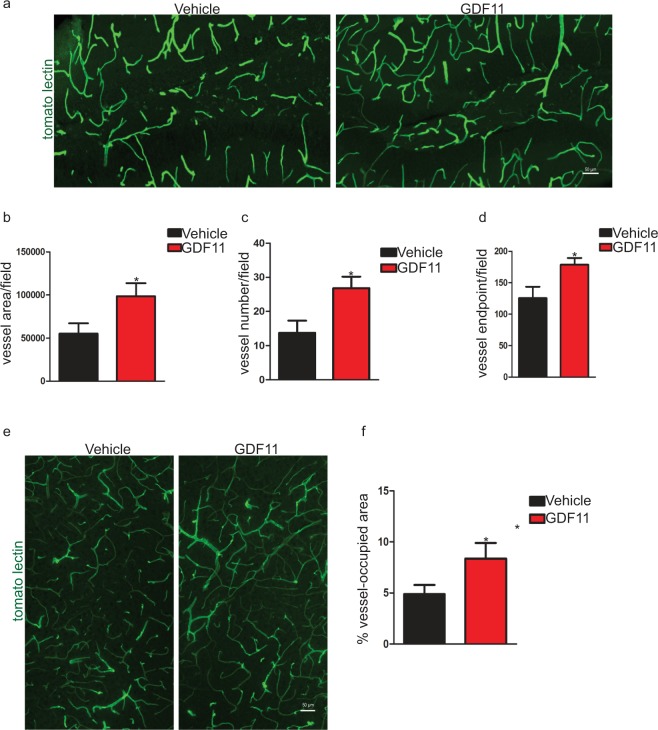
Systemic GDF11 treatment improves vasculature in the hippocampus and cortex of old mice. (**a**) Representative confocal images showing the effects of systemic GDF11 treatment on blood vessels in the dentate gyrus of old mice. (**b**) Measurement of blood vessel-occupied area (per field of view). n = 7 for each experimental group. Data shown as mean ± s.e.m., statistical analysis by unpaired, two-tailed Student’s *t-*test, *p = 0.05 compared to vehicle control. (**c**) Measurement of number of blood vessels (per field of view). n = 7 for each experimental group. Data shown as mean ± s.e.m., statistical analysis by unpaired, two-tailed Student’s *t-*test, *p = 0.02 compared to vehicle control. (**d**) Measurement of number of blood vessel endpoints (per field of view). n = 7 for each experimental group. Data shown as mean ± s.e.m., statistical analysis by unpaired, two-tailed Student’s *t-*test, *p = 0.03 compared to vehicle control. (**e**) Representative confocal images showing the effects of systemic GDF11 treatment on blood vessels in the frontal cortex of old mice. (**f**) Measurement of blood vessel-occupied area (per field of view). n = 8 for each experimental group. Data shown as mean ± s.e.m., statistical analysis by unpaired, two-tailed Student’s *t-*test, *p = 0.04 compared to vehicle control.

Aging results in impairments in structure, function and plasticity in the cerebral vasculature^7,8,19^. Given the important role blood vessels play in neurogenesis^2,3^ and our previous findings in the SVZ^11^, we speculated that systemic GDF11 treatment also could improve impaired vasculature in the hippocampus of old mice (Supplementary Fig. S2a). We used fluorescently labeled tomato lectin^20^, which almost completely overlaps (Supplementary Fig. S2b) with the known endothelial cell marker CD31^21^, to visualize changes in hippocampal vasculature with both aging and GDF11 treatment. As we expected, GDF11 increased the blood vessel-occupied area (Fig. 2a,b), number of blood vessels (Fig. 2c) and blood vessel branching (Fig. 2d) in the dentate gyrus. Again, GDF11-treated young mice did not show significant changes in any of these parameters in the dentate gyrus (Supplementary Fig. S1c–f), suggesting that angiogenic effects of systemic GDF11 treatment are also age-dependent. In addition to finding effects on hippocampal vasculature, we observed beneficial cerebrovascular effects of systemic GDF11 treatment in non-neurogenic regions of the brain such as the frontal cortex of old mice (Fig. 2e,f). This suggests that GDF11 might have a broader influence on overall CNS function in older brains via changes in the cerebral vasculature.

### Systemic GDF11 treatment increases neuronal activity markers in the hippocampus and cortex of old mice

**Figure 3:**
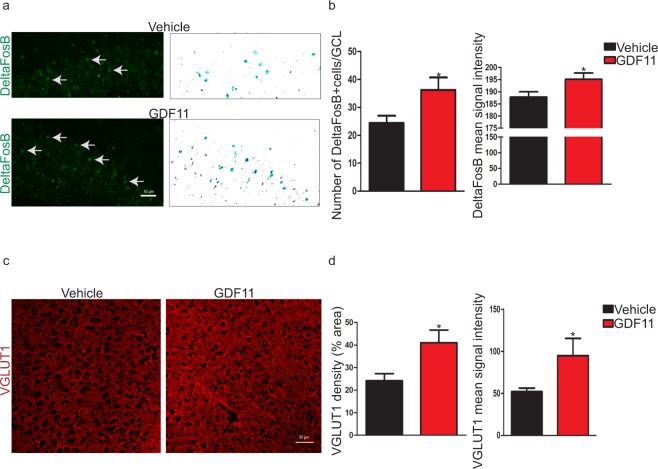
Systemic GDF11 treatment increases neuronal activity markers in the hippocampus and cortex of old mice. (**a**) Representative confocal images showing the effects of systemic GDF11 treatment on DeltaFosB levels in the GCL of old mice. White arrows indicate representative cells that are positive for the marker. Corresponding thresholded images with counted cells highlighted in blue are shown on the right. (**b**) Quantification of DeltaFosB^+^ cells and measurement of DeltaFosB mean signal intensity per cell in GCL (total area). n = 8 for each experimental group. Data shown as mean ± s.e.m., statistical analysis by unpaired, two-tailed Student’s *t-*test, *p = 0.04 compared to vehicle control. (**c**) Representative confocal images showing the effects of systemic GDF11 treatment on VGLUT1 levels in the frontal cortex of old mice. (**d**) Measurement of % VGLUT1^+^ area and VGLUT1 mean signal intensity (per field of view). n = 4 for each experimental group. Data shown as mean ± s.e.m., statistical analysis by unpaired, two-tailed Student’s *t-*test, *p = 0.04 compared to vehicle control.

Neurovascular coupling, the tight co-regulation of neuronal activity and structural and functional aspects of the cerebral vasculature, is altered with aging^22^. To evaluate whether improved vasculature in GDF11-treated old mice (Fig. 2) could also potentially translate into increased neuronal activity, we measured DeltaFosB levels in the hippocampus of old mice. DeltaFosB is a known indirect marker of long-term neuronal activity changes in response to repeated stimuli^23^. GDF11 increased the number of DeltaFosB^+^ cells and DeltaFosB mean signal intensity in the dentate gyrus, suggesting changes in neuronal activity (Fig. 3a,b). To determine whether systemic GDF11 treatment might increase excitatory neurotransmission, we measured VGLUT1 levels in the frontal cortex of old mice. VGLUT1 is a transporter highly enriched in cortex and is responsible for glutamate release at excitatory synapses^24^. GDF11 increased the level and mean signal intensity of VGLUT1 in the frontal cortex (Fig. 3c,d). GDF11 also modestly, yet non-significantly, increased synaptophysin mean signal intensity in the frontal cortex of old mice (Supplementary Fig. S3), suggesting a potential effect of GDF11 on synaptic density, similar to what had been previously shown in rat cortical neuron cultures^25^.

### Systemic GDF11 treatment slightly reduces body weight and increases food intake

Sustained, continuous exposure of mice to more than 1,000-fold the normal circulating level of GDF11 by viral delivery of a constitutively expressed construct results in significant weight loss^26^. In contrast, our daily treatment with 1 mg/kg GDF11 caused an approximately 11% reduction in body weight after one week, which plateaued for the rest of the treatment period, and the body weights between the vehicle and GDF11 groups were not significantly different at the end of the 28-day treatment (Supplementary Fig. S4a). Interestingly, GDF11-treated mice exhibited increased food intake compared to the vehicle treatment (Supplementary Fig. S4b). It is possible, therefore, that GDF11 can have some overall influence on metabolic phenotypes observed *in vivo*.

### Systemic GDF11 treatment does not affect reactive astrocytes or microglia in the hippocampus of old mice

Given the important role neuroinflammation plays in adult neurogenesis^27^, we also investigated whether systemic GDF11 treatment might have affected the number of reactive astrocytes or microglia as a potential mechanism underlying increased hippocampal neurogenesis. GDF11-treated old mice did not show increased GFAP levels (Supplementary Fig. S4c,d), a known marker of astrogliosis^28^, or Iba1 levels (Supplementary Fig. S4e,f), a known marker of reactive microglia^29^, in the dentate gyrus.

### Multiple types of analyses show that GDF11 does not cross the BBB

**Figure 4:**
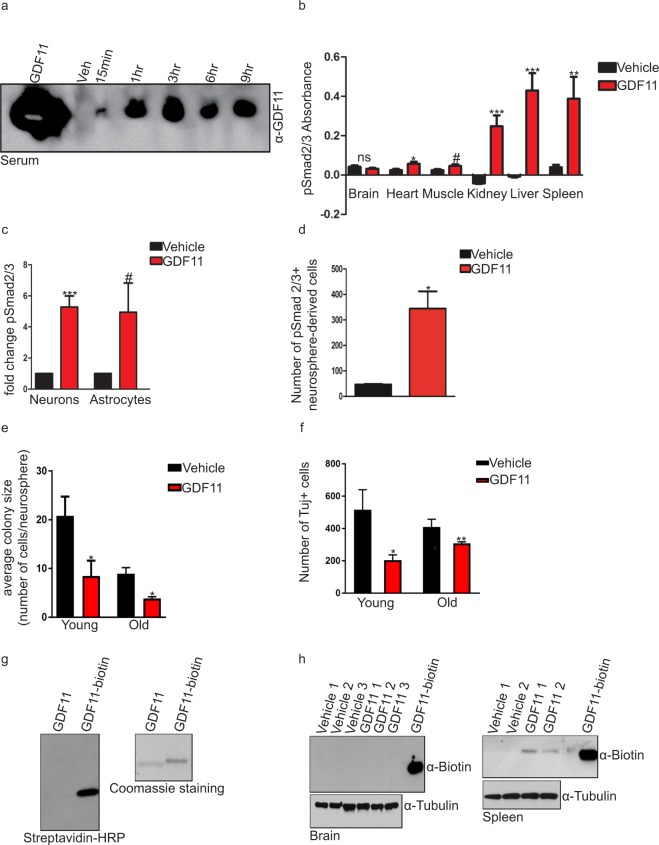
Multiple types of analyses show that GDF11 does not cross the BBB. (**a**) GDF11 levels in the serum of 24-month-old mice following acute GDF11 treatment. Full-length blot is presented in Supplementary Fig. 7a. (**b**) ELISA of SMAD2/3 phosphorylation in whole tissue lysates of 24-month-old mice following acute GDF11 treatment (1 mg/kg). n = 6 for each experimental group. Data plotted as the background-subtracted absorbance and shown as mean ± s.e.m., statistical analysis by unpaired, two-tailed Student’s *t-*test, *p = 0.03 (heart), ***p = 0.0003 (kidney), ***p = 0.0006 (liver), **p = 0.01 (spleen), ^#^p = 0.06 (muscle), not significant (ns) (brain) compared to vehicle controls of each tissue type. (**c**) ELISA of SMAD2/3 phosphorylation in primary mouse cortical neurons and primary mouse astrocytes following 1-hour treatment with GDF11 (50 ng/ml) or vehicle. n = 6 for each neuron condition, n = 4 for each astrocyte condition. Data calculated as fold change from vehicle controls and shown as mean ± s.e.m., statistical analysis by unpaired, two-tailed Student’s *t-*test, ***p = 0.0001 (neuron), ^#^p = 0.06 (astrocyte), compared to vehicle controls of each cell type. (**d**) Number of pSMAD2/3^+^ nuclei in SVZ-derived, dissociated neurospheres following 90-minute treatment with GDF11 (50 ng/ml) or vehicle. n = 3 for each condition. Data shown as mean ± s.e.m., statistical analysis by unpaired, two-tailed Student’s *t*-test, *p = 0.01 compared to vehicle control. (**e**) Average colony size (number of cells per sphere) of young and old SVZ-derived neurospheres following 10-day treatment with GDF11 (50 ng/ml) or vehicle, in a clonal assay. n = 11 for young, n = 3 for old condition. Data shown as mean ± s.e.m., statistical analysis by unpaired, two-tailed Student’s *t*-test, *p = 0.02 compared to vehicle control. (**f**) Number of Tuj1+ cells differentiated from young and old SVZ-derived neurospheres following 7-day treatment with GDF11 (50 ng/ml) or vehicle, in a differentiation assay. n = 4 for each condition. Data shown as mean ± s.e.m., statistical analysis by unpaired, two-tailed Student’s *t*-test, *p = 0.04, **p = 0.006 compared to vehicle control. (**g**) Detection of biotinylated recombinant GDF11 with streptavidin-HRP (left) or Coomassie staining (right). Full-length blot and gel are presented in Supplementary Fig. 7b. (**h**) Biotinylated GDF11 levels in the brain parenchyma (left) and the spleen (right) of 3–4-month-old mice following acute GDF11 treatment (8 mg/kg). Biotinylated recombinant GDF11 protein was loaded to help detect the biotinylated protein in tissue samples. Tubulin was used as a loading control. Full-length blots are presented in Supplementary Fig. 7c.

Whether systemically delivered GDF11 crosses the BBB and stimulates cells in the CNS directly was unknown. To test for its CNS penetration, we first treated 24-month-old mice with a single, acute i.p. injection of GDF11 or vehicle, harvested tissues, and assayed the phosphorylation of SMAD2 and SMAD3, which should increase in response to GDF11 exposure. We did not detect a significant change in pSMAD2/3 levels in whole brain even though GDF11 rapidly entered the blood (Fig. 4a) and all peripheral tissues we tested responded (Fig. 4b). This could not be not explained by the lack of responsiveness of CNS cells to GDF11 treatment. We observed increased pSMAD2/3 levels in cultures consisting either of neurons, astrocytes or neural stem cells (NSCs) following direct GDF11 treatment (Fig. 4c,d). In addition, the possibility that GDF11 does not enter the CNS is supported by the finding that *in vitro* treatment of adult NSCs with GDF11 inhibits their proliferation (Fig. 4e) and differentiation into the neuronal lineage (Fig. 4f). This is in contrast to GDF11’s pro-proliferation and differentiation effects measured after systemic administration of GDF11 (Fig. 1), but in agreement with GDF11’s endogenous roles in neurodevelopment^30^. Finally, to directly evaluate whether GDF11 crosses the BBB and to be able to trace exogenously administered GDF11, we labeled recombinant GDF11 with biotin (Fig. 4g), gave 3–4-month old mice a single, acute, intravenous (i.v.) injection of biotinylated GDF11 or vehicle, harvested the brain parenchyma and peripheral tissues, and looked for the presence or absence of biotinylated protein. Even though we could detect biotinylated GDF11 in the spleen, a peripheral tissue that does not have a microvascular barrier and was used as a positive control, we did not detect it in the brain (Fig. 4h). As a positive control, we used biotinylated transferrin (Supplementary Fig. S5a), known to be transported across the BBB^31^, and detected it in the brain parenchyma after a single, i.v. injection (Supplementary Fig. S5b). As a negative control, we used biotinylated albumin (Supplementary Fig. S5c), known to be BBB impermeable^32^, and failed to detect it in the brain parenchyma (Supplementary Fig. S5d). As the BBB may be compromised with aging, we repeated the same experiment in 19–21-month-old mice. We again were able to detect biotinylated GDF11 in the spleen, but not in the brain parenchyma (Supplementary Fig. S6a). As with young mice, biotinylated transferrin was detected in both the brain parenchyma and the spleen, while biotinylated albumin was only detected in the spleen (Supplementary Fig. S6b). Taken together, these data show that GDF11 circulating in the blood does not cross the BBB. Instead, systemically delivered GDF11’s effects on neurogenesis and neuronal function are likely indirect.

### Endothelial cells of the cerebral vasculature are responsive to GDF11

**Figure 5:**
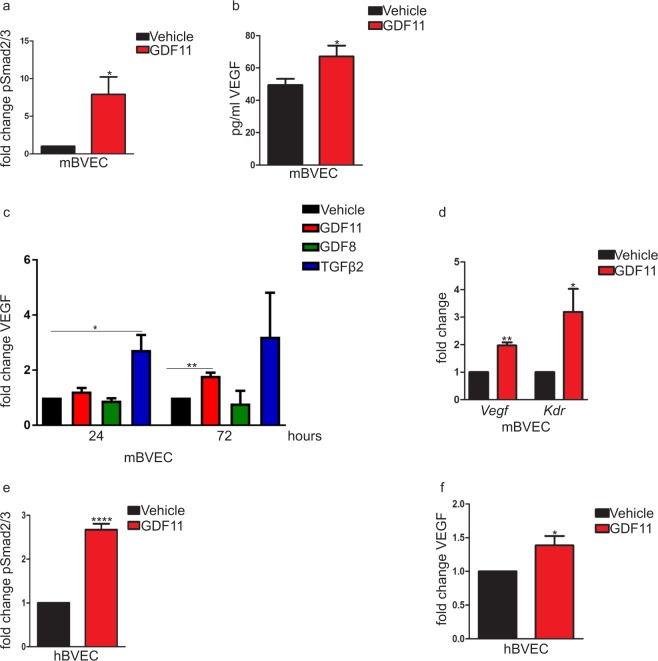
Endothelial cells of the cerebral vasculature are responsive to GDF11. (**a**) ELISA of SMAD2/3 phosphorylation in mBVECs following 1-hour treatment with GDF11 (50 ng/ml) or vehicle. n = 4 for each condition. Data calculated as fold change from vehicle control and shown as mean ± s.e.m., statistical analysis by unpaired, two-tailed Student’s *t-*test, *p = 0.03 compared to vehicle control. (**b**) ELISA of serum VEGF levels following systemic GDF11 or vehicle treatment in old mice. n = 8 for each experimental group. Data shown as mean ± s.e.m., statistical analysis by unpaired, two-tailed Student’s *t-*test, *p = 0.03 compared to vehicle control. (**c**) ELISA of VEGF levels in mBVEC conditioned medium following 24- or 72-hour treatment with GDF11 (50 ng/ml), GDF8 (50 ng/ml), TGFβ2 (50 ng/ml) or vehicle. n = 3 for each condition. Data calculated as fold change from vehicle controls and shown as mean ± s.e.m., statistical analysis by unpaired, two-tailed Student’s *t-*test *p = 0.02, **p = 0.002 compared to vehicle control at designated time point. (**d**) qPCR analysis of *Vegf* and *Kdr* gene expression following 72-hour treatment of mBVECs with GDF11 (50 ng/ml) or vehicle. n = 3 for each condition. Data calculated as fold change from vehicle controls and shown as mean ± s.e.m., statistical analysis by unpaired, two-tailed Student’s *t-*test, **p = 0.001 (*Vegf*) and *p = 0.05 (*Kdr*) compared to vehicle controls of each gene. (**e**) ELISA of SMAD2/3 phosphorylation in hBVECs following 1-hour treatment with GDF11 (50 ng/ml) or vehicle. n = 4 for each condition. Data calculated as fold change from vehicle control and shown as mean ± s.e.m., statistical analysis by unpaired, two-tailed Student’s *t-*test, ****p = 0.0001 compared to vehicle control. (**f**) ELISA of VEGF levels in hBVEC conditioned medium following 48-hour treatment with GDF11 (50 ng/ml) or vehicle. n = 6 for each condition. Data calculated as fold change from vehicle control and shown as mean ± s.e.m., statistical analysis by unpaired, two-tailed Student’s *t-*test, *p = 0.02 compared to vehicle control.

Given the apparent impermeability of BBB to GDF11 (Fig. 4h), the rapid absorption of GDF11 into the blood (Fig. 4a), and the robust changes we observed in the cerebral vasculature in response to systemic GDF11 treatment^11^ (Fig. 2), we hypothesized that endothelial cells that line the luminal surface of vessels are a target of GDF11. Although we did not detect increased pSMAD2/3 levels in whole brain following acute administration of GDF11 (Fig. 4b), endothelial cells constitute a small percentage of cells in the CNS^33^ and their response could have been obscured by relatively high baseline pSMAD2/3 signaling in whole brain (due to the endogenous CNS expression of GDF11 and other TGFβ’s)^34^. Furthermore, endothelial cells of the cerebral vasculature express the GDF11 receptor ALK5^35^. To explore the possibility that these cells are, in fact, responsive to circulating GDF11, we first tested whether brain vascular endothelial cells (BVECs) respond to GDF11. We cultured primary mouse BVECs *in vitro*, treated them with GDF11 or vehicle, and measured pSMAD2/3 levels. GDF11 increased the levels of pSMAD2/3, demonstrating that these cells are responsive and confirming our previous observations^11^ (Fig. 5a).

Vascular endothelial growth factor (VEGF) is a secreted angiogenic protein that also enhances hippocampal neurogenesis through its receptor VEGFR2 (also known as KDR or Flk-1)^36–38^. We hypothesized that GDF11 could be inducing VEGF secretion from endothelial cells as one mechanism for both the improved vasculature (Fig. 2) and the increased neurogenesis (Fig. 1) observed with systemic GDF11 treatment in old mice. To assess whether systemic GDF11 treatment affects VEGF secretion *in vivo*, we measured serum VEGF levels in old mice at the end of the GDF11 treatment. Concomitant with cerebrovascular improvements, GDF11 increased serum VEGF levels significantly (Fig. 5b). To test whether GDF11 affects VEGF levels in the brain endothelial cells *in vitro*, we treated mouse BVECs directly with GDF11, and the closely related ligands GDF8 and TGFβ2. GDF11 increased VEGF secretion while GDF8, the member of the TGFβ family with the highest sequence homology to GDF11^39^, did not change VEGF levels (Fig. 5c). TGFβ2, a more potent activator of pSMAD2/3 in BVECs and known to induce VEGF *in vitro*^40^, also increased VEGF secretion, as we expected (Fig. 5c). In addition to inducing VEGF secretion, GDF11, similar to TGFβ2^40^, increased the expression of both *Vegf* and *Kdr* (coding for VEGFR2) genes in BVECs (Fig. 5d). Increased SMAD2/3 phosphorylation and VEGF secretion following GDF11 treatment were recapitulated in primary human BVECs (Fig. 5e,f).

## Discussion

Aging is the primary risk factor for developing the most common neurodegenerative and neurovascular diseases including Alzheimer’s disease (AD), Parkinson’s disease, vascular dementia and stroke^41^. Decline in neurogenesis^42^ and degeneration of the cerebral vasculature^43^ are two of many changes that occur in the CNS with aging. Our lab previously showed that systemic treatment with GDF11, a circulating factor found in both young and old mice, enhances neurogenesis and stimulates vascular remodeling in the SVZ of old mice^11^. However, whether systemic GDF11 treatment exerts similar beneficial neurogenic and angiogenic effects in the hippocampus, whose structure and function are directly associated with cognition and negatively impacted with aging^44^, remained unknown. Furthermore, whether GDF11 achieves its diverse positive effects regardless of age had not been addressed in our previous work.

In this study, we first focused on the neurogenic and angiogenic effects of systemically delivered GDF11 in the hippocampus of both young and old mice. We demonstrate that GDF11 improves the neurovascular niche in old, but not in young, mice. Systemic GDF11 treatment increased newborn neuron (BrdU^+^/NeuN^+^), neural stem cell (Sox2^+^) and neural progenitor/immature neuron (DCX^+^) populations in the hippocampus of old mice. GDF11-treated old mice also displayed structural improvements in hippocampal vasculature. In particular, there was an increase in the number of blood vessels and the extent of vessel branching in the hippocampus following systemic GDF11 treatment. Why this treatment only works in older mice will be a topic for additional study although it might be that the normal circulating levels of GDF11 in young mice are sufficient.

Recently, there have been conflicting results regarding the role of GDF11 in aging heart and skeletal muscle^45–49^. However, our initial report^11^, studies from four independent labs^50–53^, and work presented here consistently support the conclusion that GDF11 has a positive influence on the aging CNS. Furthermore, since multiple other circulating factors have been shown to affect CNS function in old mice, there seems to be little doubt that the CNS is a surprisingly responsive target for blood-borne factors.

The fact that we observed positive effects on both neurogenesis and blood vessel integrity in the hippocampus is consistent with our prior observations relating to GDF11’s effects in the SVZ. These results are not surprising since neurogenesis is known to be controlled, at least in part, by factors associated with blood vessels^2,3^. Our data indicate that in the old mouse CNS systemic GDF11 treatment increases the level of DeltaFosB, an indirect marker of long-term neuronal activity important for regulation of hippocampal synaptic plasticity^54^, and the level of VGLUT1, which functions in glutamate release and transport essential for excitatory neurotransmission^55^. A prior study observed improved hippocampal synapse structure and function in old heterochronic parabionts^10^. It will be informative to assess whether GDF11 alone could produce similar changes. Whether GDF11-treated old mice exhibit improved cognition through olfactory discrimination tasks and/or hippocampus-dependent spatial navigation tasks also needs further research. Notably, recent studies have shown improved neurocognitive behavior in rodents treated with GDF11 in stroke^50,53^ and AD^52^ models. Therefore, it is feasible that systemic GDF11 treatment also could lead to enhanced cognition in aging.

Vascular pathology is evident in several rodent models of AD^56,57^. Neurovascular deficits including the BBB dysfunction, reduced blood flow and compromised structure of the endothelial cells that make up the BBB underlie and significantly contribute to the manifestation of neurodegenerative diseases^43,58^. Such vascular defects have been associated with neuronal atrophy, synaptic loss, cognitive impairment, and Aβ plaque accumulation^59^. Noteworthy is that our data also demonstrate that systemic GDF11 treatment improves vasculature in the frontal cortex of old mice and increases the expression of neuronal activity markers. Based on our previous work, we expect that improved blood flow will accompany the larger number of blood vessels, which we will investigate further in our future studies^11^. Because it is known that cerebral vasculature and neuronal activity are tightly co-regulated^60^ and impaired with aging, some of the positive actions of GDF11 may be attributable to processes other than neurogenesis. This is noteworthy since recent reports have cast some doubt on the magnitude of ongoing neurogenesis in the adult human brain^61^.

Perhaps most unexpectedly, our results support the hypothesis that GDF11 does not cross the BBB in appreciable quantities. Thus, the CNS effects that were observed following systemic GDF11 treatment in old mice are likely indirect, with brain endothelial cells being a potential GDF11 cellular target. In the cerebrovascular endothelium, GDF11-induced changes could occur as a consequence or combination of increased blood flow throughout the CNS, regulation of the systemic production of other circulating pro-neural factors capable of crossing the BBB, and/or by their own production of neuroactive factors. In support of the latter possibility, GDF11 treatment induced the secretion of VEGF, as well as the upregulation of *Vegf* and *Kdr* mRNA, in cultured BVECs. VEGF has been shown to both stimulate endothelial cell proliferation and enhance neurogenesis through VEGFR2^62^. It is plausible that GDF11 also regulates the production and the secretion of other CNS active factors from the brain endothelial cells, a topic we are actively pursuing.

Following the discovery of the positive CNS effects of infusing young blood into old mice, or of heterochronic parabiosis, multiple individually CNS-active factors in the circulation have been identified. Surprisingly, each one appears to have different actions and potentially different cellular targets, indicating that CNS function is determined by the additive positive and negative actions of these individual signaling molecules. Of those discovered to date, GDF11 appears to be the only one that regulates CNS function indirectly through the cerebral vasculature^4^. Our data and that of other investigators point to the existence of a complex signaling network that communicates between the brain and other tissues, is capable of regulating tissue homeostasis in aging, and may lead to the development of a new generation of effective treatments for CNS disorders.

## Methods

### Animal care

Young (2–4-month-old), middle-aged (9–11-month-old) and old (72-week-old) C57Bl/6 male mice were obtained from Jackson Laboratories and Charles River Laboratories. Pregnant C57BL/6 and CD1 female mice were obtained from Charles River Laboratories. Mice were maintained on a 12-hour light/12-hour dark cycle in a temperature controlled barrier facility, with *ad libitum* access to water and standard chow. Old mice were aged until 84-weeks-old in the facility. Young (2–3-month old) and old (22–23-month-old) mice were singly housed prior to the long-term treatment. All animal care protocols and procedures were approved by Harvard University Institutional Care and Use Committee and performed in accordance with institutional and regulatory guidelines.

### Recombinant ligands

For *in vivo* experiments, recombinant human GDF11 (Peprotech) was reconstituted in a sterile solution of 1 mM HCl in 0.5X DPBS (GIBCO). For *in vitro* experiments, recombinant human GDF11, TGFβ2 (R&D Systems or Peprotech) and GDF8 (Peprotech) were reconstituted in a sterile solution of 4 mM HCl and 0.1% BSA in 0.5X DPBS (GIBCO).

### GDF11, Bromodeoxyuridine (BrdU) and tomato lectin administration

For long-term treatment, young (2–3-month-old) and old (22–23-month-old) mice were given single i.p. injections of GDF11 (1 mg/kg) or vehicle (1 mM HCl, 0.5X DPBS) daily for 28 days. Body weights were measured weekly to adjust the dose. Food intake was measured weekly at 1, 3, 6, and 24 hours. Old mice were given i.p. injections of BrdU (50 mg/kg) twice daily for the first 3 days of the long-term treatment. For the terminal procedure, mice were anesthetized with Avertin and given a single, i.v. injection of DyLight® 488 labeled tomato lectin (100 µg) (Vector Laboratories)^20^. 5 minutes following tomato lectin injection, mice were perfused transcardially with 20 ml of ice-cold 1X DPBS followed by 20 ml of ice-cold 4% PFA in 1X DPBS. Brains were removed, post-fixed in 4% PFA in 1X DPBS overnight at 4 °C, switched to PBS (with salt concentrations of 136.9 mM sodium chloride, 2.7 mM potassium chloride, 10.1 mM sodium phosphate and 1.1 mM potassium phosphate) with sodium azide and kept at 4 °C until further processing.

### Immunostaining

Each mouse brain was embedded in 3% agarose and 50 µm-thick, free-floating coronal sections were cut in the Leica VT1000S vibrating microtome. Sections were kept in PBS with sodium azide at 4 °C until immunostaining. Sections were permeabilized and blocked in 10% normal goat or donkey serum and 0.1% Triton™ X-100 in PBS for 1 hour at room temperature. For BrdU immunostaining, sections were pre-treated with 2 N HCl for 30 minutes at 37 °C prior to permeabilization and blocking steps. Sections were incubated overnight at 4 °C with the following primary antibodies at 1:100 dilution in blocking solution: rat monoclonal anti-BrdU (ab6326, Abcam), rabbit polyclonal anti-Sox2 (2748, Cell Signaling Technology), goat polyclonal anti-DCX (8066, Santa Cruz Biotechnology), mouse monoclonal anti-NeuN (MAB377, EMD Millipore), rabbit monoclonal anti-FosB (2251, Cell Signaling Technology), rabbit polyclonal anti-VGLUT1 (135 303, Synaptic Systems), rabbit monoclonal anti-synaptophysin (ab16659, abcam), rat monoclonal anti-CD31 (550274, BD Biosciences), chicken polyclonal anti-GFAP (4674, Abcam), rabbit polyclonal anti-Iba1 (019-19741, Wako). Neurosphere-derived cells were permeabilized and blocked in 10% normal goat or donkey serum, 5% BSA and 0.1% Triton™ X-100 in PBS for 1 hour at room temperature and incubated overnight at 4 °C with mouse monoclonal anti-pSMAD2/3 (610842, BD Biosciences) primary antibody at 1:5,000 dilution or mouse monoclonal anti-Tuj1 (MMS-435P, BioLegend) primary antibody at 1:2,000 dilution in blocking solution. Alexa Fluor® 488, 568, 647 secondary antibodies were used at 1:500 dilution in 1% normal goat or donkey serum in PBS for 1 hour at room temperature. Hoechst 33342 was used to label nuclei.

### Imaging equipment and settings

In all *in vivo* staining experiments, images were acquired using the Zeiss ELYRA superresolution confocal microscope with EC Plan-Neofluar lenses at 10X and 20X magnification, MBS 488/561/633 beam splitters, PMT detectors and EM-CCD camera (Andor iXon). Excitation and emission spectral range of the channels were the following: Alexa Fluor® 488 (494–582); Alexa Fluor® 568 (572–650); Alexa Fluor® 647 (638–755). Acquired images were at 1024 × 1024 pixel resolution with an average of 2–4 lines, 8 bit image depth, and z-stacks of 1 µm intervals. Images were processed as maximum intensity projections of acquired z-stacks. Images were acquired and visualized using the Zeiss Zen black software. Imaging and analysis were performed blinded: each animal was assigned a code that was revealed after the analysis. For each experimental group, 6–8 mice were used unless stated otherwise. For each mouse, 3–4 bregma-matched sections comprising the dentate gyrus or the frontal cortex were imaged. Image analysis was done using the ImageJ software and thresholding for cell counting, mean signal intensity and %area/density measurements, and the AngioTool software for blood vessel measurements. For *in vitro* staining experiments, imaging was performed using the Operetta high content imaging microscope (Perkin Elmer) at 10X magnification. Images were acquired and visualized using the Harmony software (Perkin Elmer). For each individual well of the 96-well plates, half of the field was imaged. Image analysis was done using the Columbus image analysis software (Perkin Elmer) for nuclei counting.

### GDF11 biotinylation

Recombinant human GDF11 was incubated with a 50-fold molar excess of EZ-Link Sulfo-NHS-LC-biotin reagent (ThermoFisher Scientific) in 1 mM sodium hydroxide at room temperature for 2.5 hours. The reaction was quenched by adding ethanolamine to a final concentration of 2 mM. The solution was diluted to 15 mL in 10 mM HCl, and removal of unreacted biotin, buffer exchange, and protein concentration was achieved by Amicon® Ultra-4 Ultracel 3 K MWCO centrifugal filtration. Serial dilution and concentration steps were used to step the HCl down to a final concentration of 2 mM.

### Blood brain barrier penetration

Young (3–4-month-old) mice were given a single i.v. injection of a molar-equivalent dose of biotinylated GDF11 (8 mg/kg), transferrin (25 mg/kg) (Sigma Aldrich) or albumin (22.5 mg/kg) (Sigma Aldrich). Old (19–21-month-old) mice were given a single i.v. injection of a molar-equivalent dose of biotinylated GDF11 (1 mg/kg or 8 mg/kg), transferrin (3 mg/kg) or albumin (3 mg/kg). 3–4 hours following biotinylated protein injection, mice were perfused transcardially with 20 ml of ice cold 1X DPBS. Brain tissue was collected to isolate the brain parenchyma as described below. Liver and spleen were collected as peripheral tissue controls. Tissues were homogenized in Pierce™ RIPA lysis buffer with Halt™ protease and phosphatase inhibitors using the T 10 BASIC ULTRA-TURRAX^®^ tissue homogenizer. Homogenates were centrifuged at 10,000xg for 10 minutes, and protein extracts were frozen at −80 °C until further processing. Protein concentrations were assessed using the Pierce™ BCA Protein Assay Kit (ThermoFisher Scientific).

### Parenchyma isolation

Brain parenchyma was depleted of capillaries and isolated following previously published protocols^63,64^. Briefly, brain tissue was dissected and dounce homogenized in Hank’s buffered salt solution with 20 mM HEPES at pH = 7.4. Homogenate was spun at 1,000 × g for 5 minutes, and the lipid and capillary pellet was discarded. Concentrated RIPA lysis buffer was added to the supernatant, homogenate was incubated on ice for 15–30 minutes, spun at 10,000 × g for 10 minutes, and parenchymal supernatant was frozen at −80 °C until further processing.

### Immunoblotting

Biotinylated protein levels in mouse tissues were assessed by immunoblotting. Tissue lysates were denatured at 95 °C for 5 minutes in 4X Laemmli sample buffer with 10% β-mercaptoethanol. Samples were loaded on Criterion™ TGX™ any KD gels and run in Novex® Tris-Glycine SDS running buffer. Samples were then transferred to Trans-Blot^®^ Turbo™ Midi PVDF using the Bio-Rad rapid semi-dry system. After Ponceau S staining, membranes were blocked in 2% non-fat milk in Tris-buffered saline with Tween-20^®^ for 1 hour at room temperature. Membranes were incubated overnight at 4 °C with the following primary antibodies at 1:5,000 dilution in blocking solution: Rabbit polyclonal anti-biotin (ab1227, Abcam), rabbit polyclonal anti-transferrin (ab82411, Abcam), rabbit polyclonal anti-BSA (A11133, ThermoFisher Scientific), rabbit polyclonal anti-GAPDH (ab9485, Abcam), rabbit polyclonal anti-beta tubulin (ab6046, Abcam). Goat anti-rabbit HRP secondary antibody or Pierce™ high sensitivity streptavidin-HRP antibody (21130, ThermoFisher Scientific) was added at 1:10,000 dilution in blocking solution and membranes were incubated for 1 hour at room temperature. Signal was visualized using SuperSignal™ West Dura Extended Duration Substrate or SuperSignal™ West Femto Maximum Sensitivity Substrate on HyBlot CL^®^ film.

### GDF11 immunoblotting

GDF11 protein levels in mouse serum were assessed by immunoblotting. Blood was collected and allowed to clot in SST-Amber Microtainer™ tubes, spun at 2,000 × g for 10 minutes, and serum was collected and frozen at −80 °C until further processing. For immunoblotting, 5–10 µl of mouse serum was denatured at 70 °C for 10 minutes in NuPAGE™ LDS sample buffer with fresh reducing agents (100 mM dithiothreitol and 5% β-mercaptoethanol). Samples were loaded on 4–12% NuPAGE™ bis-tris gradient protein gels and run in NuPAGE™ 1X MES SDS running buffer supplemented with antioxidants (5 mM sodium metabisulfite and NuPAGE™ antioxidant). Samples were then transferred to Trans-Blot^®^ Turbo™ Mini PVDF using the Bio-Rad rapid semi-dry system. After Ponceau S staining, membranes were blocked in 5% non-fat milk in Tris-buffered saline with Tween-20^®^ for 1 hour at room temperature. A mouse monoclonal primary antibody selective for GDF11 over GDF8 (MAB19581, R&D Systems) was added at 1:1,000 dilution in blocking solution and incubated overnight at 4 °C. Heavy chain-specific goat anti-mouse HRP secondary antibody (ab98693, Abcam) was added at 1:5,000 dilution in blocking solution and membranes were incubated for 1 hour at room temperature. Signal was visualized using SuperSignal™ West Dura Extended Duration Substrate on BIOMAX film.

### Primary neuronal cultures

Primary mouse neurons were derived from E16-E17 CD1 mouse cortices via standard protocols. Embryonic tissue was dissected, digested with papain, and dissociated by trituration in the presence of DNAse I. Papain was quenched with ovomucoid albumin inhibitor and cells were collected via filtration and centrifugation. Neurons were plated on tissue culture dishes coated with poly-D-lysine and laminin, and maintained in Neurobasal^®^ medium supplemented with B27^®^, glutamine, and penicillin/streptomycin for 9–18 days before use. Cells were treated with GDF11 (50 ng/ml) or vehicle (4 mM HCl, 0.1% BSA, 0.5X DPBS) for 1 hour prior to pSMAD2/3 analysis.

### Primary astrocyte cultures

Primary mouse astrocytes were derived from P1-P4 C57BL/6 mouse cortices via standard protocols. Postnatal tissue was dissected, digested with 0.25% trypsin, dissociated by trituration in the presence of DNAse I, and cells were collected via filtration and centrifugation. Astrocytes were plated on tissue culture dishes coated with poly-D-lysine and maintained in Dulbecco’s Modified Eagle Medium (DMEM) supplemented with 10% fetal bovine serum, glutamine, and penicillin/streptomycin. Once the cells reached 70–90% confluency, they were passaged and maintained on gelatin coated plastic. Cells were treated with GDF11 (50 ng/ml) or vehicle (4 mM HCl, 0.1% BSA, 0.5X DPBS) for 1 hour prior to pSMAD2/3 analysis.

### Neural stem cell cultures

Neurosphere cultures were generated by microdissection of SVZ from young and old mice and maintained as free-floating spheres on ultra-low adhesion plastic in DMEM/F12 supplemented with B27 without vitamin A^®^, penicillin/streptomycin and 20 ng/ml epidermal growth factor and fibroblast growth factor 2^65^. To assess for *in vitro* responsiveness to GDF11, neurospheres were dissociated with Accutase^®^ according to the manufacturer’s instructions and allowed to adhere to Matrigel^®^-coated plates for 16 hours. Adherent cells were treated with GDF11 (50 ng/ml) or vehicle (4 mM HCl, 0.1% BSA, 0.5X DPBS) for 90 minutes prior to pSMAD2/3 analysis.

### Clonal assay

Neurospheres at passage 3–8 were dissociated using Accutase^®^ and sorted at one cell per well of 96-well round bottom, uncoated tissue culture plates. Forward scatter/side scatter criteria and Calcein Blue AM were used on the MoFLO™ XDP sorter (Beckman Coulter Life Sciences) to sort single live cells. Half of the wells were treated GDF11 (50 ng/ml), and the other half were treated with vehicle (4 mM HCl, 0.1% BSA, 0.5X DPBS). Media was changed to replace growth factors and ligands every 3–4 days. After 10 days *in vitro*, wells were assessed for the presence of spheres, and the number of cells per sphere was determined by bright field microscopy and staining with Hoechst 33342. Average colony size was compared between vehicle and GDF11-treated conditions.

### Differentiation assay

Neurospheres were maintained as described above (see Neural stem cell cultures) and treated with GDF11 (50 ng/ml) or vehicle (4 mM HCl, 0.1% BSA, 0.5X DPBS) for 7 days. After 7 days *in vitro*, neurospheres were dissociated using Accutase^®^, plated on laminin-coated plastic in serum containing differentiation media (DMEM+ 10% fetal bovine serum) for 7 days. Differentiation into the neuronal lineage was determined by immunostaining for Tuj1.

### Endothelial cell cultures

Primary mouse brain microvascular endothelial cells (mBVECs) were purchased from Cell Biologics and maintained in Complete Endothelial Cell Medium (Cell Biologics) according to the manufacturer’s instructions. mBVECs were trypsinized in 0.25% trypsin and seeded on gelatin-coated plastic. Cells were treated with GDF11 (50 ng/ml), TGFβ2 (50 ng/ml), GDF8 (50 ng/ml) or vehicle (4 mM HCl, 0.1%BSA, 0.5X DPBS) for 24 or 72 hours. Human brain microvascular endothelial cells (hBVECs) were purchased from iXCells Biotechnologies and maintained in Endothelial Cell Growth Medium (iXCells Biotechnologies) according to the manufacturer’s instructions. hBVECs were trypsinized in 0.05% trypsin and seeded on gelatin-coated plastic. Cells were treated with GDF11 (50 ng/ml) or vehicle (4 mM HCl, 0.1%BSA, 0.5X DPBS) for 48 hours. Cell culture media was collected at designated time points for mouse and human VEGF analysis.

### Enzyme-linked immunosorbent assays (ELISAs)

All collected tissue and cell samples were homogenized in Pierce^®^ RIPA lysis buffer with Halt™ protease and phosphatase inhibitors. Protein concentrations were assessed using the Pierce™ BCA Protein Assay Kit. pSMAD2/3 in tissue and cell lysates was assayed using the PathScan^®^ Phospho-SMAD2 (Ser465/Ser467)/Phospho-SMAD3 (Ser423/Ser425) Sandwich ELISA kit (Cell Signaling Technology) according to the manufacturer’s instructions. After long-term GDF11 treatment of old mice, blood was collected in BD Microtainer™ Capillary Blood Collector Tubes with K_2_EDTA, spun at 10,000 × g for 10 minutes, plasma was collected and frozen at −80 °C until further processing. VEGF in mouse serum and VEGF in cell culture media were assayed using the mouse (R&D Systems) and human (R&D Systems) VEGF Quantikine ELISA kits according to the manufacturer’s instructions.

### Gene expression

For gene expression analysis, total RNA was extracted using TRIzol^®^ and further purified with the RNeasy^®^ kit (Qiagen). cDNA was synthesized from total RNA using the iScript™ Reverse Transcription Supermix (Bio-Rad). Quantitative real-time PCR was carried out using the Fast SYBR^®^ Green Master Mix (ThermoFisher Scientific) and cell lysates were run using the QuantStudio™ 12 K Flex Real Time PCR System (ThermoFisher Scientific). The housekeeping gene *Hprt1* was used as an internal control. Primers used were the following: *Vegf* (PPM03041F, ThermoFisher Scientific), *Kdr* (P_f_: TTTCACCTGGCACTCTCCAC P_r_: CCCCTTGGTCACTCTTGGTC, *Hprt1* (PPM03559F, ThermoFisher Scientific).

### Statistical analysis

All statistical analyses were performed using the GraphPad Prism software. Normal distribution of the data was tested with Shapiro-Wilk normality test. Results were expressed as mean ± s.e.m. Comparisons between groups were made by unpaired, two-tailed Student’s *t*-test or two-way ANOVA, as appropriate. Power calculations were carried out using the PS software and the sample size was determined based on power of at least 80% and error rate of 5% in unpaired Student’s *t*-test. Statistical significance was designated with *p < 0.05; **p < 0.01, ***p < 0.001, ****p < 0.0001. Detailed statistical analyses are described in the Figure Legends.

## Electronic supplementary material

Supplementary Information

## Data Availability

All data generated or analyzed in this study are included in this published article and its Supplementary Information files.
