# Supplementary material for: Growth Differentiation Factor 11 treatment leads to neuronal and vascular improvements in the hippocampus of aged mice
Source: Sci Rep. 2018 Nov 23;8:17293. doi: 10.1038/s41598-018-35716-6 (PMC6251885; doi:10.1038/s41598-018-35716-6)
Supplement: Supplementary file 1 — Supplementary Information [file 41598_2018_35716_MOESM1_ESM.pdf]

Growth Differentiation Factor 11 treatment leads to neuronal and vascular improvements in the hippocampus of aged mice.

Ceren Ozek[1,2,*], Richard C. Krolewski[1,2,3], Sean M. Buchanan[1,2,#], Lee L. Rubin[1,2,*,#]

Author Affiliations: [1]Department of Stem Cell and Regenerative Biology, Harvard University, Cambridge, MA, 02138 [2]Harvard Stem Cell Institute, Harvard University, Cambridge, MA, 02138 [3]Department of Neurology, Brigham and Women's Hospital, Massachusetts General Hospital, Boston, MA, 02115

Contact Information (in order): ceren_ozek@harvard.edu, rkrolewski@fas.harvard.edu, sean_buchanan@harvard.edu, lee_rubin@harvard.edu.

[*]Corresponding author

[#]These authors contributed equally to this work.

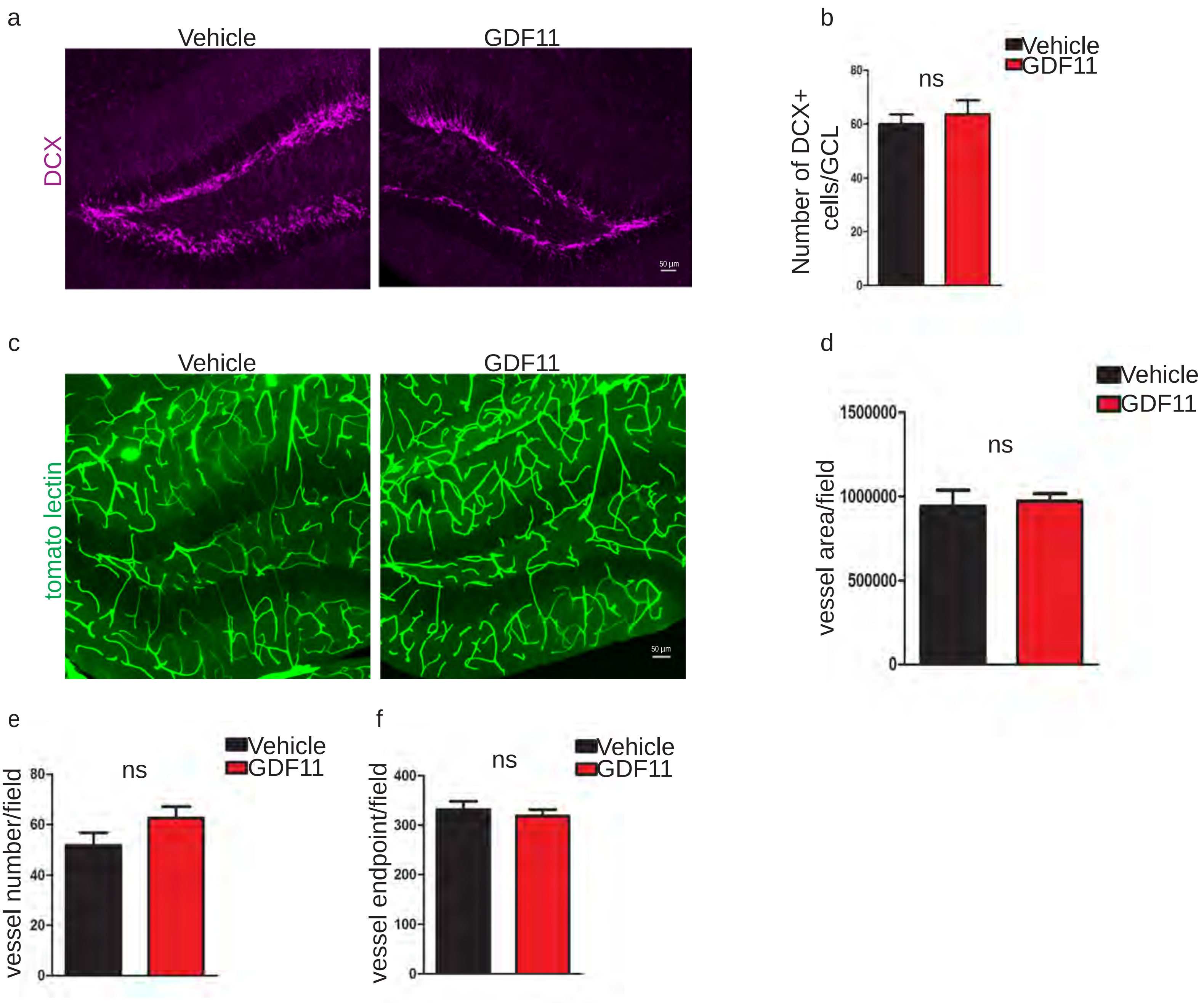

a
Vehicle
GDF11
DCX
50 µm
b
Vehicle
GDF11
ns
Number of DCX+ cells/GCL
0
20
40
60
80
c
Vehicle
GDF11
tomato lectin
50 µm
d
Vehicle
GDF11
ns
vessel area/field
0
500000
1000000
1500000
e
Vehicle
GDF11
ns
vessel number/field
0
20
40
60
80
f
Vehicle
GDF11
ns
vessel endpoint/field
0
100
200
300
400

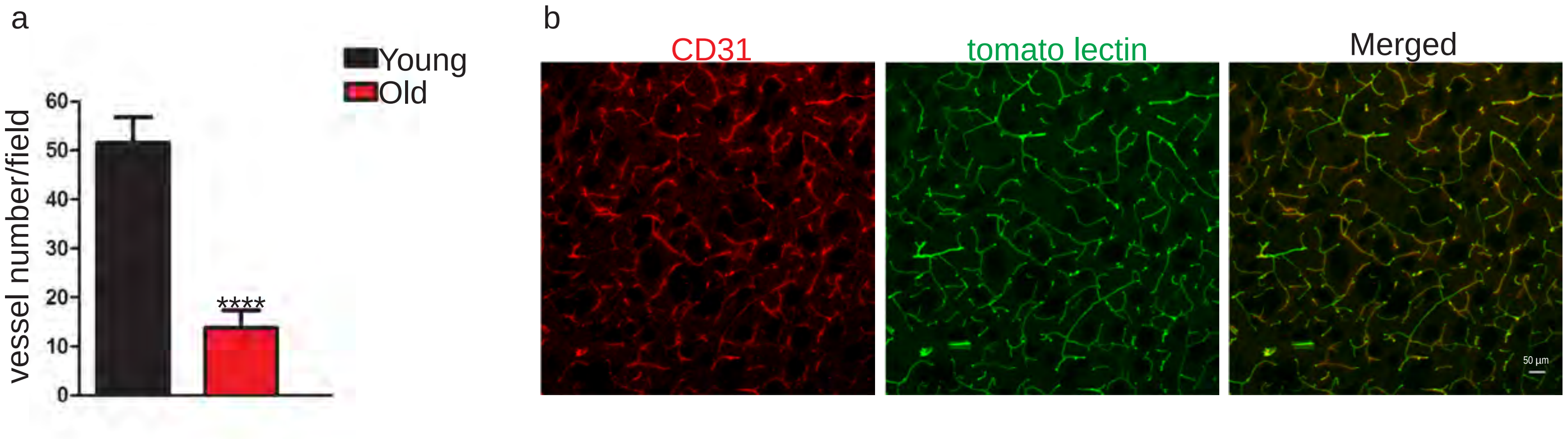
a
vessel number/field
60
50
40
30
20
10
0
****
Young
Old
b
CD31
tomato lectin
Merged
50 µm

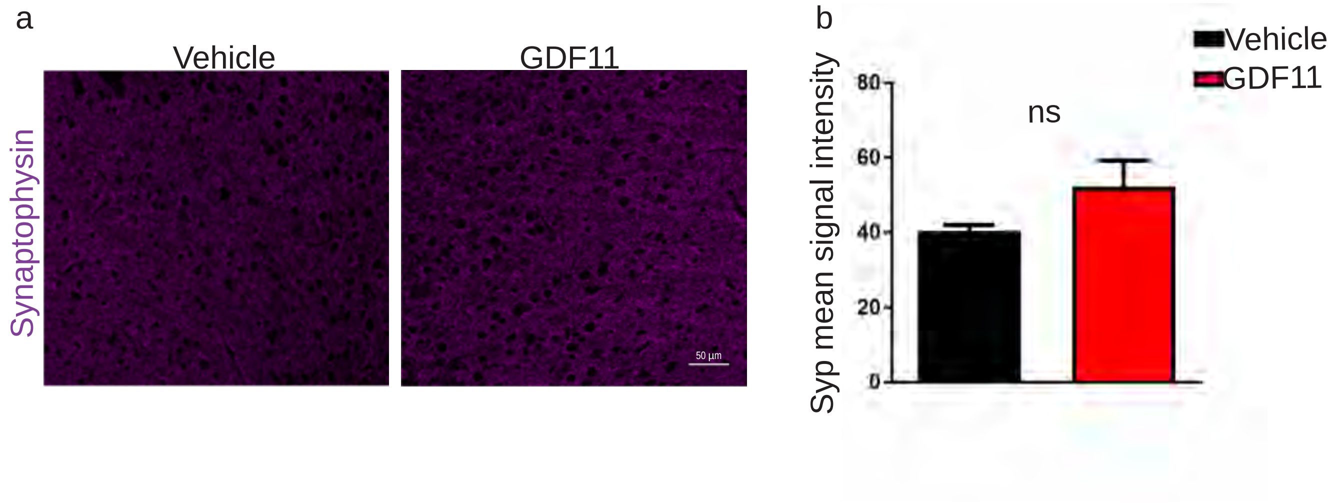

a
Vehicle
GDF11
Synaptophysin
50 µm
b
Vehicle
GDF11
Syp mean signal intensity
80
60
40
20
0
ns

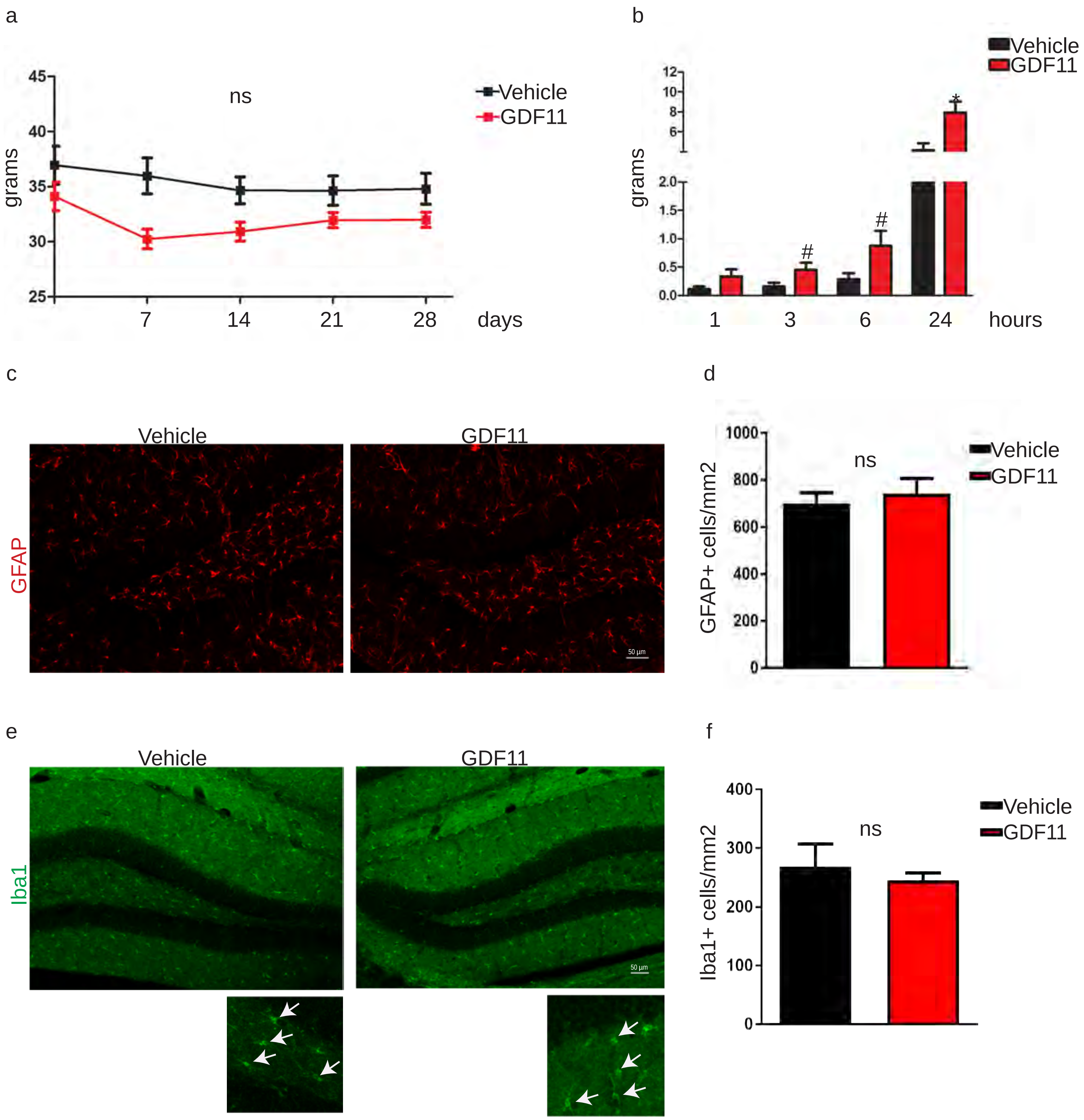

a
ns
Vehicle
GDF11
grams
45
40
35
30
25
7
14
21
28
days
b
Vehicle
GDF11
grams
12
10
8
6
2.0
1.5
1.0
0.5
0.0
#
#
*
1
3
6
24
hours
c
Vehicle
GDF11
GFAP
50 µm
d
ns
Vehicle
GDF11
GFAP+ cells/mm2
1000
800
600
400
200
0
e
Vehicle
GDF11
Iba1
50 µm
f
ns
Vehicle
GDF11
Iba1+ cells/mm2
400
300
200
100
0

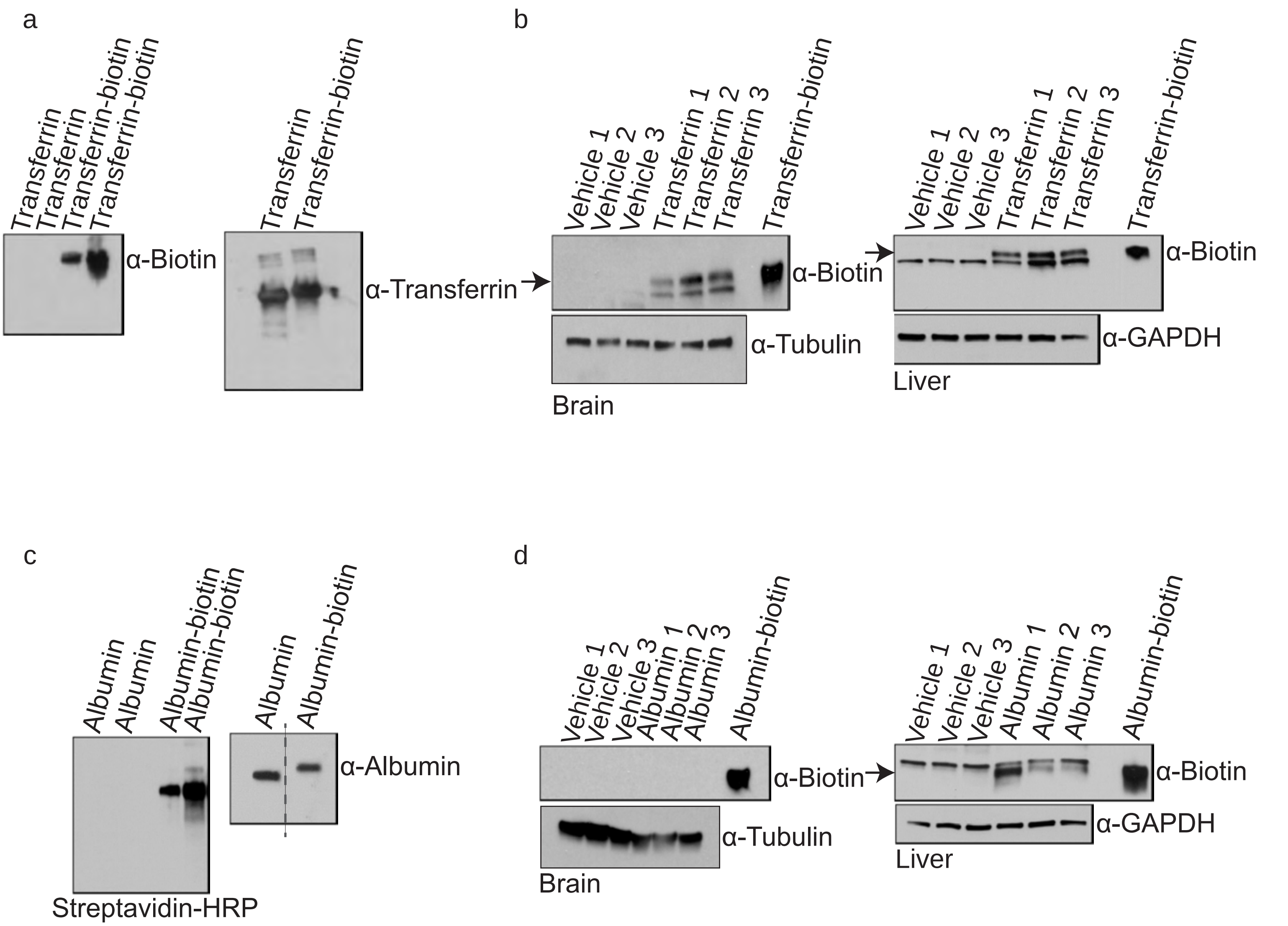
a
Transferrin
Transferrin
Transferrin-biotin
Transferrin-biotin
α-Biotin
Transferrin
Transferrin-biotin
α-Transferrin
b
Vehicle 1
Vehicle 2
Vehicle 3
Transferrin 1
Transferrin 2
Transferrin 3
Transferrin-biotin
α-Biotin
α-Tubulin
Brain
Vehicle 1
Vehicle 2
Vehicle 3
Transferrin 1
Transferrin 2
Transferrin 3
Transferrin-biotin
α-Biotin
α-GAPDH
Liver
c
Albumin
Albumin
Albumin-biotin
Albumin-biotin
Streptavidin-HRP
Albumin
Albumin-biotin
α-Albumin
d
Vehicle 1
Vehicle 2
Vehicle 3
Albumin 1
Albumin 2
Albumin 3
Albumin-biotin
α-Biotin
α-Tubulin
Brain
Vehicle 1
Vehicle 2
Vehicle 3
Albumin 1
Albumin 2
Albumin 3
Albumin-biotin
α-Biotin
α-GAPDH
Liver

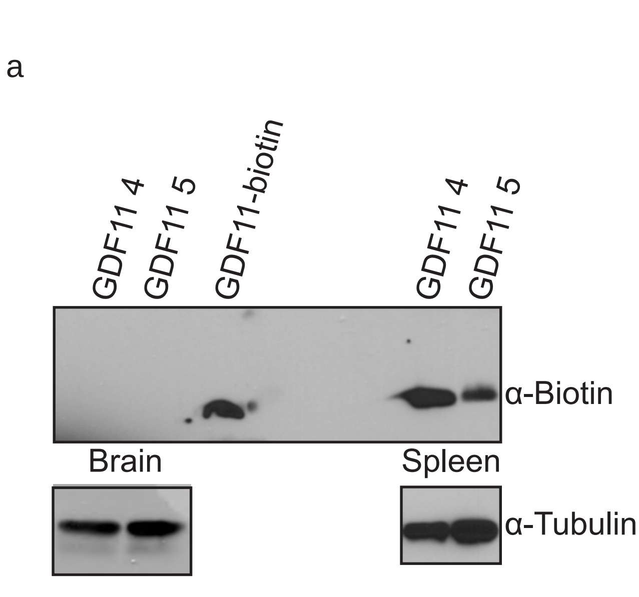
a
GDF11 4
GDF11 5
GDF11-biotin
GDF11 4
GDF11 5
α-Biotin
Brain
Spleen
α-Tubulin
b
Transferrin 3-brain
Transferrin 3-spleen
Albumin 3-brain
Albumin 3-spleen
Transferrin-biotin
Albumin-biotin
α-Biotin

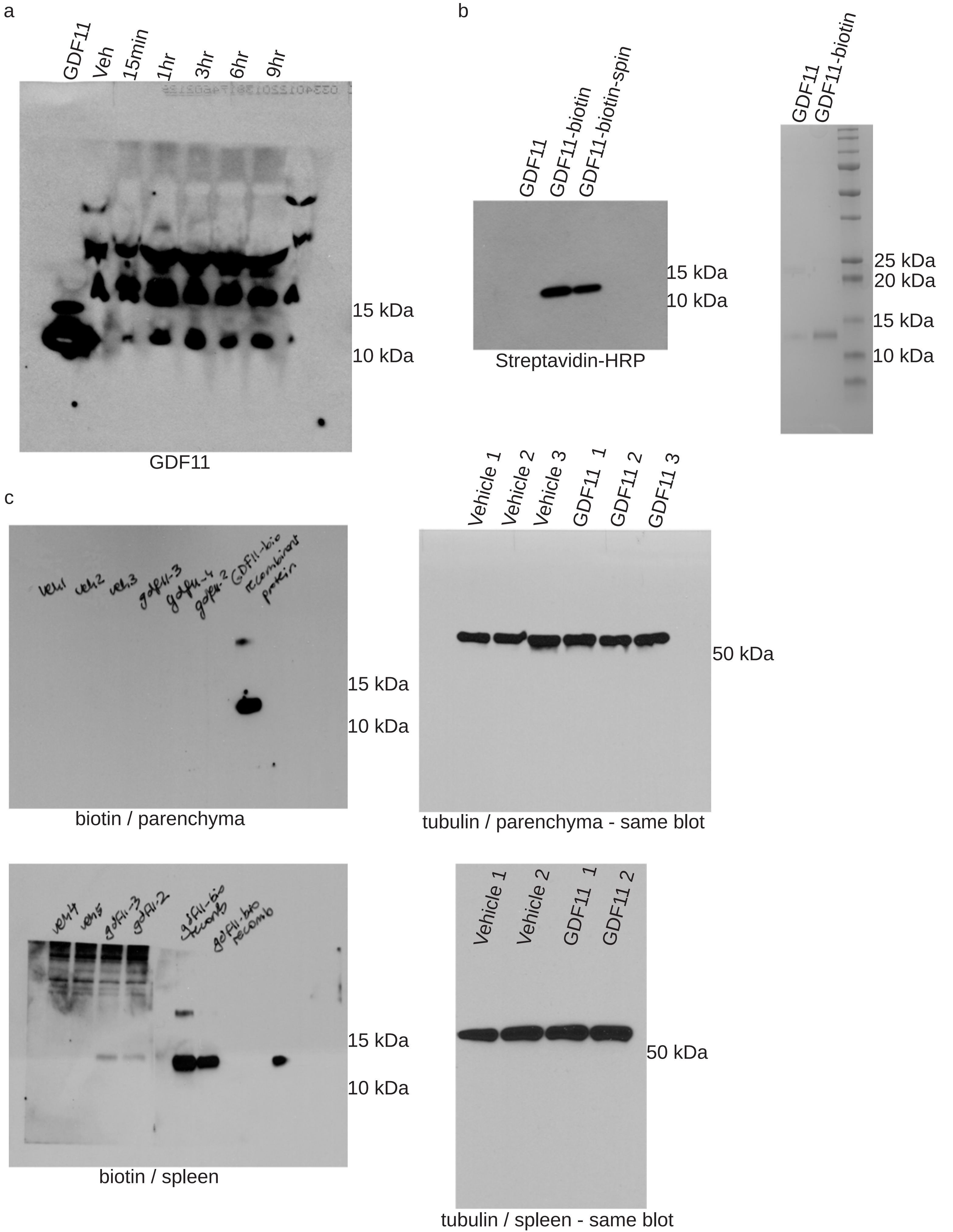
a
GDF11
Veh
15min
1hr
3hr
6hr
9hr
15 kDa
10 kDa
GDF11
b
GDF11
GDF11-biotin
GDF11-biotin-spin
15 kDa
10 kDa
Streptavidin-HRP
GDF11
GDF11-biotin
25 kDa
20 kDa
15 kDa
10 kDa
c
15 kDa
10 kDa
biotin / parenchyma
Vehicle 1
Vehicle 2
Vehicle 3
GDF11 1
GDF11 2
GDF11 3
50 kDa
tubulin / parenchyma - same blot
15 kDa
10 kDa
biotin / spleen
Vehicle 1
Vehicle 2
GDF11 1
GDF11 2
50 kDa
tubulin / spleen - same blot

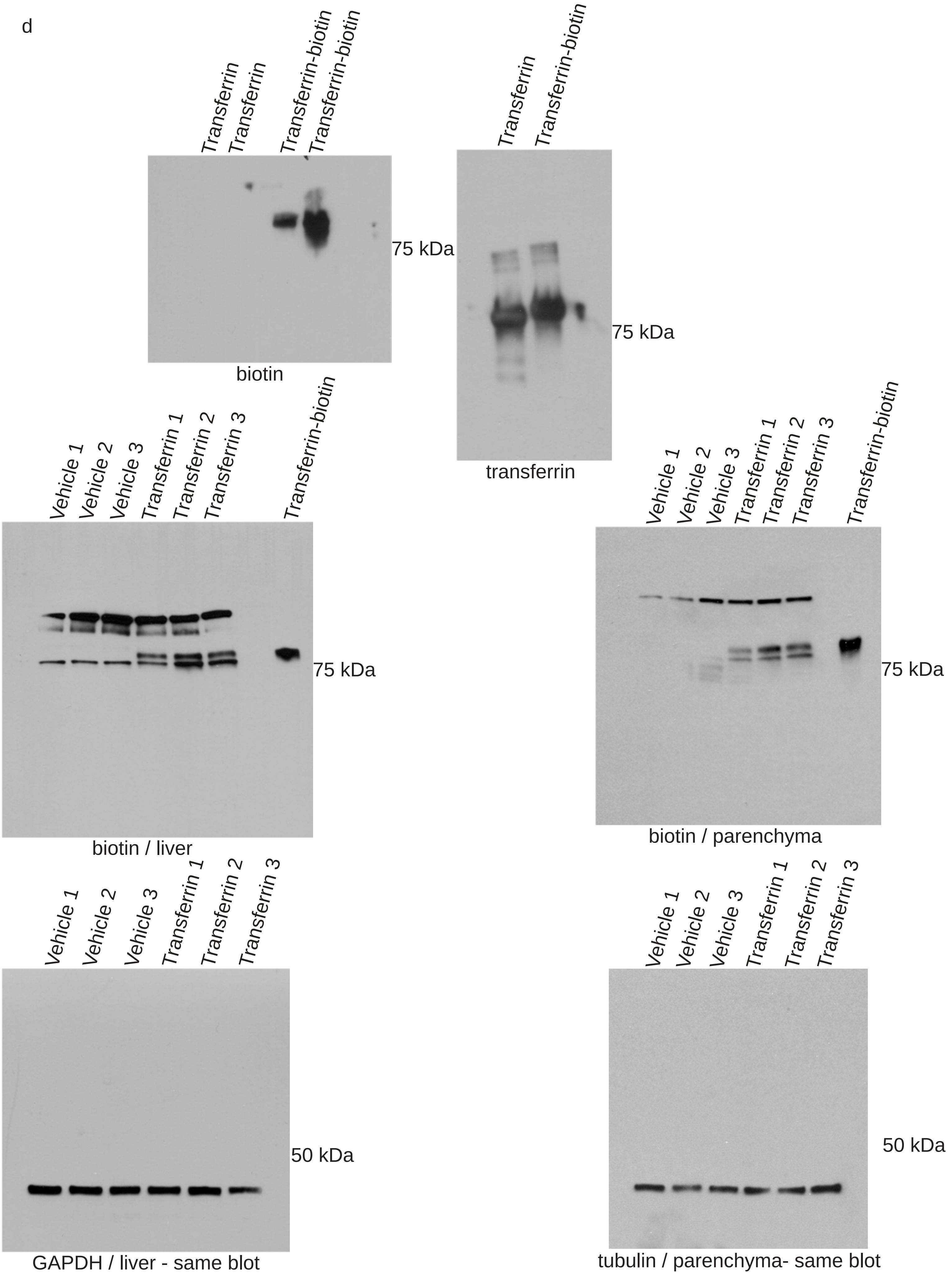

d
Transferrin
Transferrin
Transferrin-biotin
Transferrin-biotin
75 kDa
biotin
Transferrin
Transferrin-biotin
75 kDa
transferrin
Vehicle 1
Vehicle 2
Vehicle 3
Transferrin 1
Transferrin 2
Transferrin 3
Transferrin-biotin
75 kDa
biotin / liver
Vehicle 1
Vehicle 2
Vehicle 3
Transferrin 1
Transferrin 2
Transferrin 3
Transferrin-biotin
75 kDa
biotin / parenchyma
Vehicle 1
Vehicle 2
Vehicle 3
Transferrin 1
Transferrin 2
Transferrin 3
50 kDa
GAPDH / liver - same blot
Vehicle 1
Vehicle 2
Vehicle 3
Transferrin 1
Transferrin 2
Transferrin 3
50 kDa
tubulin / parenchyma- same blot

e

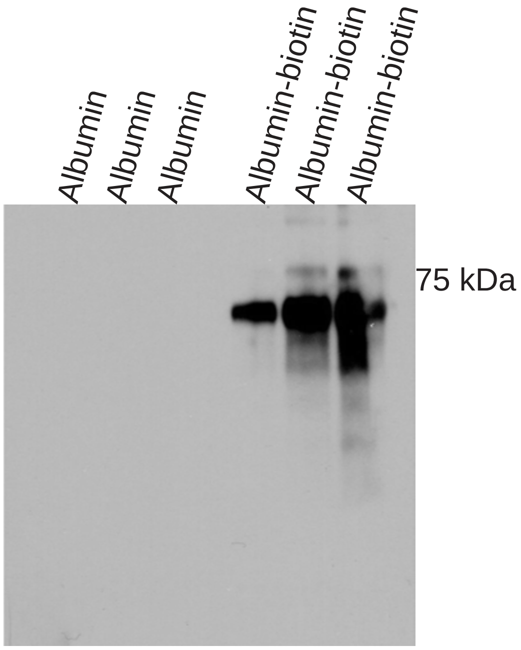

Streptavidin-HRP

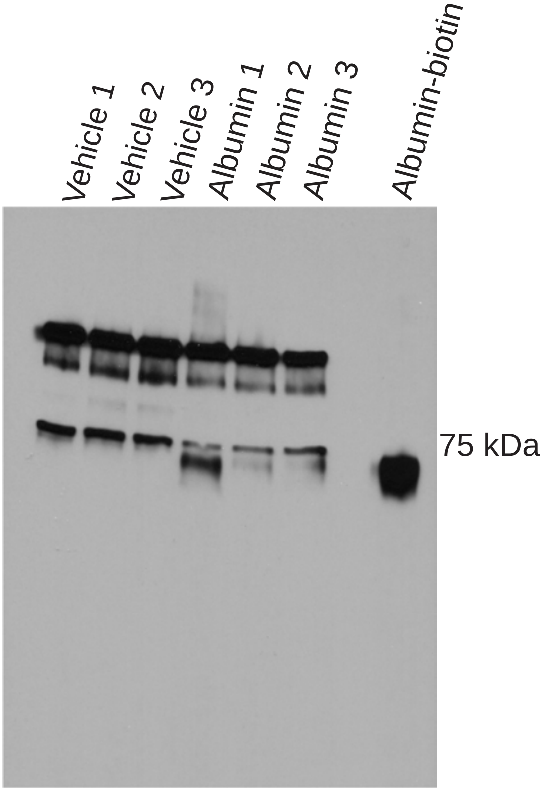

biotin / liver

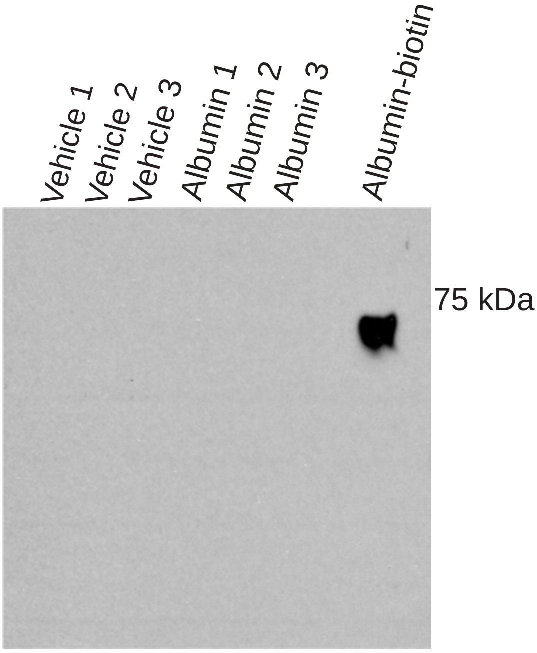

biotin / parenchyma

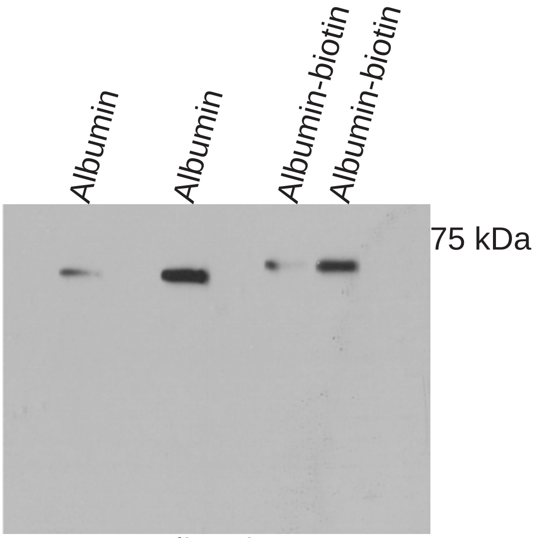

Albumin
uncropped

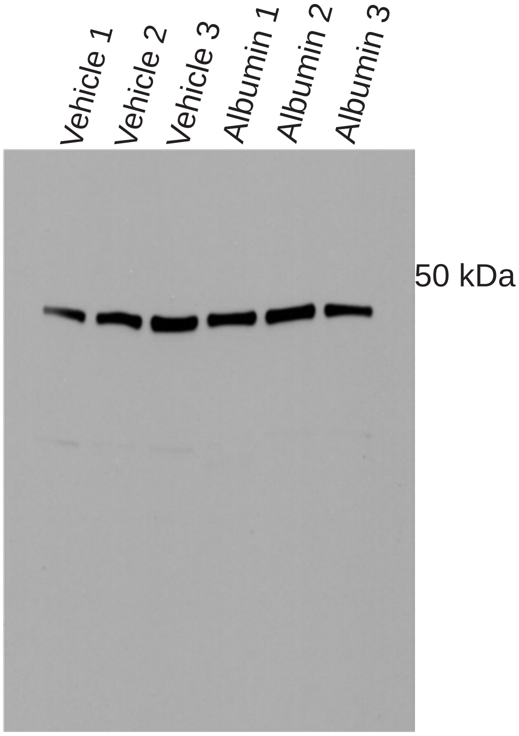

GAPDH / liver - same blot

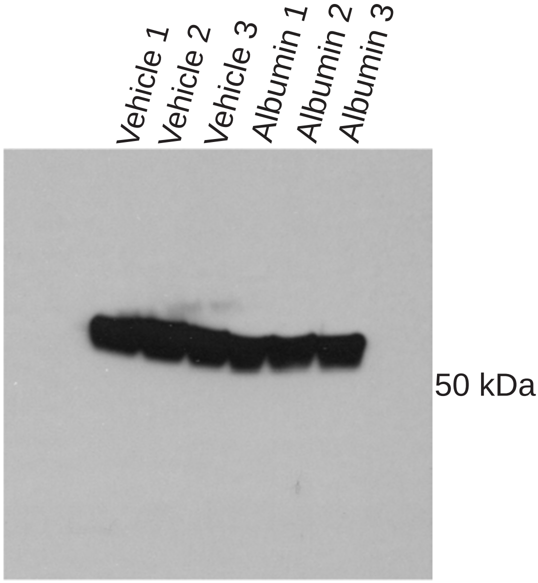

tubulin / parenchyma - same blot
darker exposure

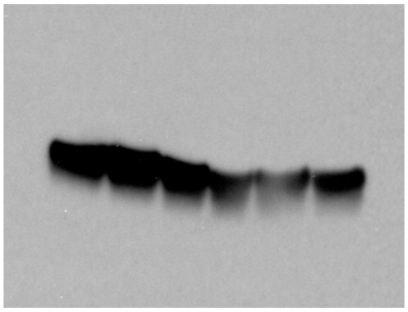

tubulin / parenchyma - same blot
lighter exposure

f

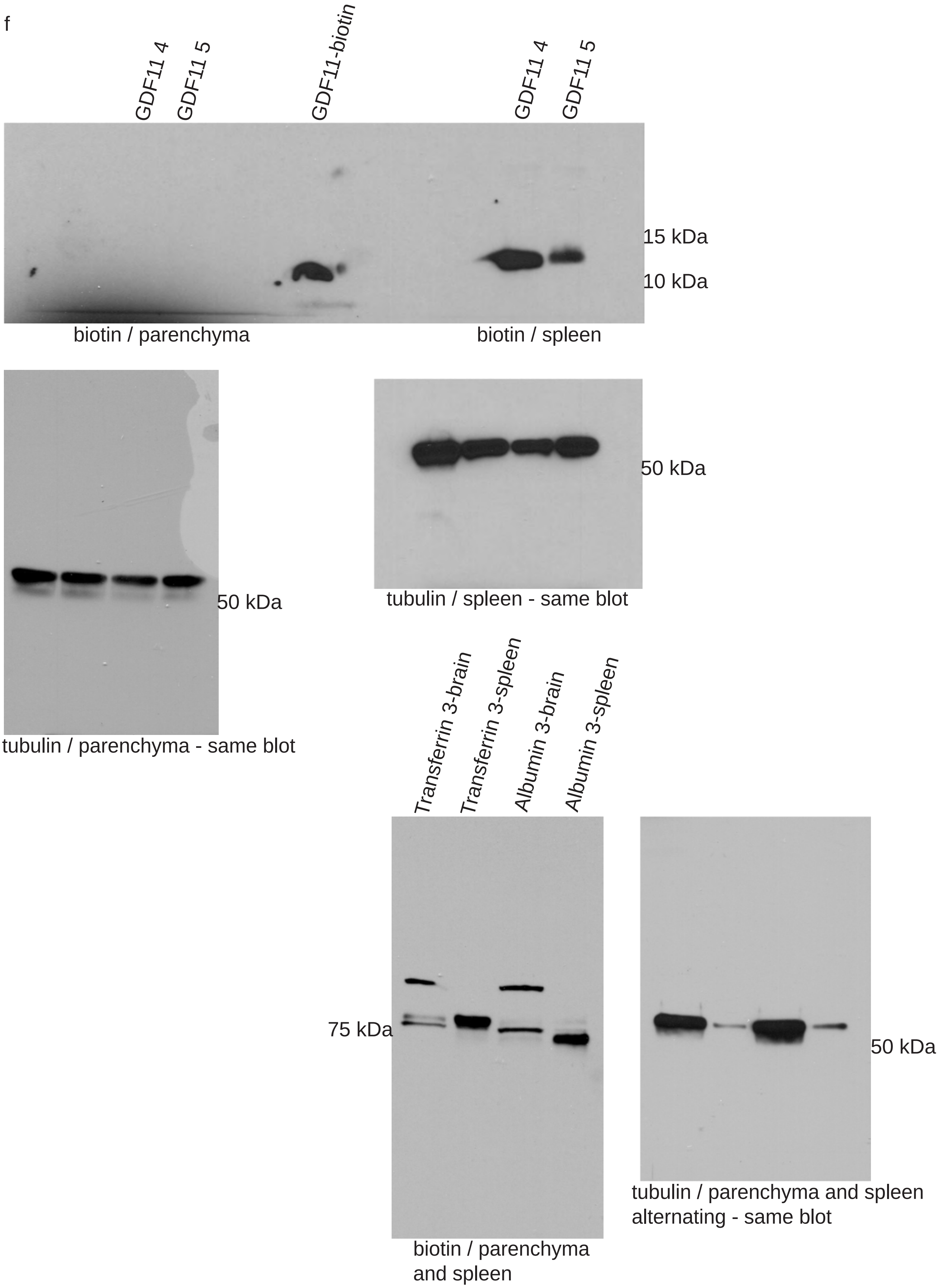
GDF11 4
GDF11 5
GDF11-biotin
GDF11 4
GDF11 5
15 kDa
10 kDa
biotin / parenchyma
biotin / spleen
50 kDa
tubulin / parenchyma - same blot
50 kDa
tubulin / spleen - same blot
Transferrin 3-brain
Transferrin 3-spleen
Albumin 3-brain
Albumin 3-spleen
75 kDa
biotin / parenchyma and spleen
50 kDa
tubulin / parenchyma and spleen alternating - same blot

Supplementary Figure 1. Systemic GDF11 treatment does not have significant neurogenic or angiogenic effects in the hippocampus of young mice.

(**a**) Representative confocal images showing the effects of systemic GDF11 treatment on DCX^+ neural progenitor/immature neurons in the GCL of young mice. (**b**) Quantification of DCX^+ neural progenitor/immature neurons in GCL (per mm^2 of total area). n=6 for each experimental group. Data shown as mean ± s.e.m., statistical analysis by unpaired, two-tailed Student's *t*-test, not significant (ns). (**c**) Representative confocal images showing the effects of systemic GDF11 treatment on blood vessels in the dentate gyrus of young mice. (**d**) Measurement of blood vessel-occupied area (per field of view). (**e**) Measurement of number of blood vessels (per field of view). (**f**) Measurement of number of blood vessel endpoints (per field of view). n=6 for each experimental group in F-H. Data shown as mean ± s.e.m., statistical analysis by unpaired, two-tailed Student's *t*-test, not significant (ns).

Supplementary Figure 2. Cerebral vasculature is impaired with aging, and tomato lectin is a reliable marker of blood vessels.

(**a**) Measurement of number of blood vessels in the dentate gyrus of young and old mice. n=6 for young mice, n=8 for old mice. Data shown as mean ± s.e.m., statistical analysis by unpaired, two-tailed Student's *t*-test, ****p=0.0001 compared to young mice. (**b**) Representative confocal images showing co-localization of CD31 and tomato lectin staining in the frontal cortex.

Supplementary Figure 3. Systemic GDF11 treatment causes a modest increase in synaptophysin levels in the cortex of old mice.

(**a**) Representative confocal images showing the effects of systemic GDF11 treatment on synaptophysin level in the frontal cortex of old mice. (**b**) Measurement of synaptophysin mean signal intensity (per field of view). n=5 for each experimental group. Data shown as mean ± s.e.m., statistical analysis by unpaired, two-tailed Student's *t*-test, not significant (ns).

Supplementary Figure 4. Systemic GDF11 treatment slightly reduces body weight and increases food intake but does not affect reactive astrocytes or microglia in the hippocampus of old mice.

(**a**) Body weight curves during systemic GDF11 or vehicle treatment in old mice. n=8 for each experimental group. Data shown as mean ± s.e.m., statistical analysis by two-way ANOVA, not significant (ns). (**b**) Cumulative food intake after one week of systemic GDF11 or vehicle treatment in old mice. n=8 for each experimental group. Data shown as mean ± s.e.m., statistical analysis by unpaired, two-tailed Student's *t*-test, *p=0.01 #p=0.06 compared to vehicle control at designated time point. (**c**) Representative confocal images showing the effects of systemic GDF11 treatment on GFAP level in the dentate gyrus of old mice. (**d**) Quantification of $GFAP^+$ astrocytes (per mm^2). n=7 for each experimental group. Data shown as mean ± s.e.m., statistical analysis by unpaired, 2-tailed Student's *t*-test, not significant (ns). (**e**) Representative confocal images showing the effects of systemic GDF11 treatment on Iba1 level in the dentate gyrus of old mice. White arrows in the insets indicate representative cells that are

positive for the marker. (**f**) Quantification of Iba1$^+$ microglia (per mm^2). n=5 for each experimental group. Data shown as mean ± s.e.m., statistical analysis by unpaired, two-tailed Student's *t*-test, not significant (ns).

Supplementary Figure 5. Biotinylated transferrin, but not biotinylated albumin, crosses the BBB.

(**a**) Detection of biotinylated recombinant transferrin with immunoblotting for biotin (left) and transferrin (right). Full-length blots are presented in Supplementary Figure 7d. (**b**) Biotinylated transferrin levels in the brain parenchyma (left) and the liver (right) of 3-4-month-old mice following acute transferrin treatment (25 mg/kg). Biotinylated recombinant transferrin protein was loaded to help detect the biotinylated protein in tissue samples. Arrow points to the specific band. Tubulin and GAPDH were used as loading controls. Full-length blots are presented in Supplementary Figure 7d. (**c**) Detection of biotinylated recombinant albumin with streptavidin-HRP (left) or by immunoblotting for albumin (right). Anti-albumin blot is cropped and stitched (dashed black line). Full-length blots are presented in Supplementary Figure 7e. (**d**) Biotinylated albumin levels in the brain parenchyma (left) and the liver (right) of 3-4-month-old mice following acute albumin treatment (22.5 mg/kg). Biotinylated recombinant albumin protein was loaded to help detect the biotinylated protein in tissue samples. Arrow points to the specific band. Tubulin and GAPDH were used as loading controls. Full-length blots are presented in Supplementary Figure 7e.

Supplementary Figure 6. Biotinylated GDF11 does not cross the BBB of old mice.

(**a**) Biotinylated GDF11 levels in the brain parenchyma (left) and the spleen (right) of 19-21-month-old mice following acute GDF11 treatment (1 or 8 mg/kg for samples 4 and 5, respectively). Biotinylated recombinant GDF11 protein was loaded to help detect the biotinylated protein in tissue samples. Tubulin was used as a loading control. Full-length blots are presented in Supplementary Figure 7f. (**b**) Biotinylated transferrin and biotinylated albumin levels in the brain parenchyma and the spleen of 19-21-month-old mice following acute transferrin or albumin treatment (3 mg/kg). Biotinylated recombinant transferrin and albumin proteins were loaded to help detect the biotinylated protein in tissue samples. Arrow points to the specific band. Tubulin was used as a loading control. Full-length blots are presented in Supplementary Figure 7f.

Supplementary Figure 7. Full-length gels and blots.

(**a**) Full-length blot of Fig. 4a. (**b**) Full-length blot and gel of Fig. 4g. (**c**) Full-length blots of Fig. 4h. (**d**) Full-length blots of Supplementary Fig. 5a,b. (**e**) Full-length blots of Supplementary Fig. 5c,d. (**f**) Full-length blots of Supplementary Fig. 6a,b.
